# Climate Change and Intertidal Wetlands

**DOI:** 10.3390/biology2010445

**Published:** 2013-03-19

**Authors:** Pauline M. Ross, Paul Adam

**Affiliations:** 1 School of Science and Health, University of Western Sydney, Hawkesbury K12, Locked Bag 1797, Penrith, Sydney, New South Wales 2751, Australia; 2 School of Biological, Earth and Environmental Sciences, University of New South Wales, Sydney, New South Wales 2052, Australia; E-Mail: p.adam@unsw.edu.au

**Keywords:** mangrove, saltmarsh, climate change, sealevel rise, ocean acidification, ocean warming, molluscs, crabs

## Abstract

Intertidal wetlands are recognised for the provision of a range of valued ecosystem services. The two major categories of intertidal wetlands discussed in this contribution are saltmarshes and mangrove forests. Intertidal wetlands are under threat from a range of anthropogenic causes, some site-specific, others acting globally. Globally acting factors include climate change and its driving cause—the increasing atmospheric concentrations of greenhouse gases. One direct consequence of climate change will be global sea level rise due to thermal expansion of the oceans, and, in the longer term, the melting of ice caps and glaciers. The relative sea level rise experienced at any one locality will be affected by a range of factors, as will the response of intertidal wetlands to the change in sea level. If relative sea level is rising and sedimentation within intertidal wetlands does not keep pace, then there will be loss of intertidal wetlands from the seaward edge, with survival of the ecosystems only possible if they can retreat inland. When retreat is not possible, the wetland area will decline in response to the “squeeze” experienced. Any changes to intertidal wetland vegetation, as a consequence of climate change, will have flow on effects to biota, while changes to biota will affect intertidal vegetation. Wetland biota may respond to climate change by shifting in distribution and abundance landward, evolving or becoming extinct. In addition, impacts from ocean acidification and warming are predicted to affect the fertilisation, larval development, growth and survival of intertidal wetland biota including macroinvertebrates, such as molluscs and crabs, and vertebrates such as fish and potentially birds. The capacity of organisms to move and adapt will depend on their life history characteristics, phenotypic plasticity, genetic variability, inheritability of adaptive characteristics, and the predicted rates of environmental change.

## 1. Introduction

Soft shore intertidal wetlands comprise a number of distinctive communities including mudflats, mangroves, saltmarsh, high level hypersaline flats (sometimes referred to as sabkha), freshwater tidal wetlands, and intergradations between them. These wetlands are found fringing estuaries (both permanent estuaries and those which are only intermittently open to the sea), but may also be found on low wave energy open coasts. In this review, we will discuss primarily saltmarshes and mangrove forests, although we recognise that there are ecological interactions between all of the intertidal wetland types. We endorse in particular the comment of Rovai *et al.* [1] that high level flats and their relationships to other communities have been the much neglected. Intertidal wetlands interact ecologically with adjacent estuarine and coastal waters, and the exchange of material, in both directions, between wetlands and adjacent waters [2,3,4,5,6,7] is a major reason why the need for protection and management of intertidal wetlands is given a high priority in many jurisdictions. Seagrass beds also occur close the intertidal wetlands and some faunal elements utilising intertidal wetlands also occur in seagrasses, but the vast majority of seagrasses are subtidal and outside the scope of this review.

One of the anticipated consequences of climate change for soft shore intertidal wetlands is sea level rise. If sedimentation fails to keep pace with sea level rise survival of the current extent of intertidal wetland communities will depend on their ability to move inland [8,9,10]. If inland movement is not possible then species unable to utilise other habitats may be at risk, at least locally, of extinction [10,11,12,13]. Restrictions to movement inland may be imposed by existing topography or by features constructed by humans, such as the presence of barriers constructed to prevent flooding, leading to what has been referred to as “coastal squeeze” [9]. The capacity of a species to keep pace with the rate of climate change will also depend on the velocity of climate change of the habitat. The velocity of climate change describes the speed at which climate is changing, being a ratio of temporal and spatial gradients of mean annual near-surface temperature (°C/year ÷ °C/km = km/year) [8]. The anticipated rate of climate change in spatially flat, flooded landscapes such as saltmarshes and mangrove forests is predicted to be the most rapid of all global ecosystems [8]. Mountainous biomes will have the slowest velocities of climate change because the spatial gradient of temperature change is greatest over modest displacements. Flooded grasslands, such as saltmarshes, and mangrove forests will have the highest velocities because the spatial gradient of temperature change is lowest over greatest displacements. This means a species in saltmarsh and mangrove forests needs to move further for temperature to change appreciably compared to species in many other biomes. It is also less feasible for species to keep pace with climate change in saltmarsh and mangrove forests because of their history of reclamation and fragmentation [2]. While intertidal wetlands may have originally had continuous distributions along the shores of many estuaries, their distribution is now often fragmented by human developments. Estuaries may be separated one from another by long stretches of coastline inhospitable to the development of intertidal wetlands so that there may be considerable barriers to migration of species in response to climate change.

The main focus of this review is to summarise predicted impacts of climate change on the flora and fauna of saltmarshes and mangrove forests identify knowledge gaps and encourage potential research to develop measures to conserve these threatened ecosystems.

## 2. Saltmarshes and Mangrove Forests

Coastal saltmarshes are intertidal ecosystems occurring on soft sediments on which the vegetation is dominated by flowering plants—graminoids, forbs and low shrubs. They can be distinguished from mangroves, which occur in a similar habitat, where the predominant vegetation comprises trees. Mangroves are mainly found on tropical and subtropical coasts, and where they extend into temperate regions, are usually stunted, sometimes to the extent that they are shorter than some of the shrubs on adjacent saltmarshes. Both saltmarshes and mangroves are most frequently found in estuaries, but can occur on open coasts if the wave energy is low [2].

Saltmarsh has a cosmopolitan distribution, being found on all continents except Antarctica. Textbooks and popular accounts often regard saltmarshes as the equivalent ecosystem in temperate regions to mangroves in the tropics, but there are areas of saltmarsh in the tropics and in Australia, for example, the area of saltmarsh north of the Tropic of Capricorn is greater than that further south. On tropical and subtropical arid shores, saltmarsh may be replaced by extensive, frequently hypersaline intertidal flats, with very scattered occurrences of flowering plants and where the surface and first centimetre or so in depth of the sediment is a mat of microorganisms and microalgae. These flats, sometimes referred to as sabhka, are productive ecosystems, and on the few occasions each year when they are subject to tidal inundation, provide pulses of nutrients and organic matter to the adjacent estuary or shallow embayment [2].

There are relatively few species of trees in mangroves, and biogeographically, they can be separated into two broad groupings: the more species-rich Indian Ocean-West Pacific region, and the much more depauperate East Pacific-Atlantic grouping. Species richness is highest at low latitudes on coasts with relatively high rainfall throughout the year; species richness declines with increasing latitude and with increasing seasonality of rainfall [14,15]. Mangroves are temperature sensitive and growth rates, reproductive success, phenology and latitudinal limits of individual species appear to be temperature controlled [16,17,18]. The sea provides an ameliorating influence on the local climate of the coast, so that the extremes of temperature experienced even a short distance inland are rare occurrences within saltmarsh and mangrove habitats [19]. In the southeastern United States, frost, which is a rare occurrence on coasts, appears to set a limit on the northern distribution of mangroves, but in Australia and New Zealand, *Avicennia marina* (Forssk.) Vierh reaches the highest latitudes of any mangrove species in the world, and at least in New Zealand, tolerates the occasional frost [2,19]. Warming, particularly in winter, may permit species to extend to higher latitudes.

The total vascular flora of saltmarshes is much larger than that of mangroves, and shows much more regional variation [2]. Unlike most other biomes, saltmarshes are distinctive in that species richness in the tropics is low, and increases in the temperate zone. Broad latitudinal bands of distinct saltmarsh floristic assemblages can be recognised, but this pattern is interrupted by the very distinctive assemblage of *Spartina*-dominated marshes on the Gulf and Atlantic coasts of North America. Less well studied than the vascular flora and the fauna are the algae, fungi and microorganisms of saltmarsh and mangroves, although it is known that they are responsible for many of the ecosystem services provided by these systems. Algae include both macroalgae (free-living and epiphytic) and the vast array of microalgae that may be important for stabilising sediment surfaces during colonisation and as a resource for a variety of grazers. Fungi are important decomposers as are bacteria [2]. The films of fungi and microorganisms that form on detritus are an important component of detrital food chains. While we might anticipate that climate change will affect individual species in the assemblages of these organisms, it is less clear how ecological processes will be affected [2].

Wetlands, in general, have an important global role in carbon sequestration and regulation of greenhouse gas emissions. Different types of wetland may be either sources or sinks of carbon. Anaerobic wetland soils provide suitable conditions for bacterial production of methane and nitrous oxide, although saline conditions inhibit methanogenesis so that intertidal wetlands produce less methane than freshwater wetlands. Drainage of wetlands can result in loss of carbon through oxidation and release of carbon dioxide. Mangroves sequester large amounts of carbon, and accumulation of organic matter below ground is an important mechanism by which mangroves can adjust to rising sea level [5,7,20,21,22]. Saltmarshes may also accumulate organic matter in sediment, particularly in *Spartina* marshes, but in some marshes inorganic sedimentation prevails [4]. As well as accumulating organic matter in sediment, intertidal wetlands export organic matter, both particulate and dissolved, into adjacent waters and also provide habitat for a great diversity of organisms including many commercially important fishery species [2,5,7,23].

The substrate on which saltmarsh and mangroves develop varies considerably and ranges from coarse calcareous deposits through sand to silt and fine clays. Many saltmarsh and mangrove species appear to be tolerant of a wide range of sediment conditions, although a small number may favour, for example, sand over clay [2,24]. Tidal flooding interacts with sediment type to create gradients of drainage and oxygenation status of the sediment which profoundly influences local distribution of species within individual sites [2]. Waterlogging of the sediment not only affects the amount of oxygen available to roots and to the infauna, it also affects the oxidation state of a variety of anions so that, for example, in more waterlogged sites iron is present in the ferrous state, and manganese in the manganous state; these reduced ionic forms are generally more phytotoxic than the oxidised states. Sulfur is present as sulfide and a strong smell of hydrogen sulfide is characteristic of many saltmarsh and mangrove sediments. The majority of flooding-tolerant plants are not capable of significant growth under anoxic conditions [25], but maintain an internal aerobic state through development of extensive aerenchyma which is important not only to the plants themselves but also modifies the soil environment [26]. In general, the microenvironment surrounding roots in anaerobic soils is ameliorated by leakage of oxygen from aerenchyma within the roots [27]; saltmarsh plants and mangroves frequently have extensive aerenchyma and other root modifications. The oxidised zone immediately adjacent to the roots provides a specialised habitat for the infauna (and in particular the often abundant meiofauna). *Spartina alterniflora* is the overwhelmingly dominant species on vast areas of saltmarsh in the Gulf and Atlantic coasts of North America. In better-drained parts of the marsh, the well-developed aerenchyma, which provides a continuous space between shoots and roots, permits aerobic respiration. However, under poor drainage conditions, the aerenchyma is insufficient to support complete aerobic respiration [28]. Survival is possible through a high rate of alcoholic fermentation, with toxic ethanol diffusing out of the roots. This may involve carbon loss through accelerated glucose production (which is fermented to produce ethanol) and from the loss of ethanol into the soil [28]. Other species with similar physiological features would also incur a cost in terms of carbon loss in order to survive anaerobic conditions, but Hovenden *et al.* [29] showed that in *Avicennia marina* seedlings the aerenchyma has insufficient storage capacity to maintain aerobic conditions if there is complete submergence for more than three to five hours and this could be a factor determining the seaward limit of the species, which would thus retreat if sea level rises. As climate change continues, the effects of increased temperature on respiration rates and fermentation may add to the stresses on wetland plants.

The sediment type also interacts with tidal inundation and climatic conditions to determine the salinity to which organisms are exposed. At the lowest levels on the shore to which saltmarsh or mangrove extend, flooding is a regular, almost daily occurrence (although even at these levels flooding is normally for less than half the day). Higher up the shore, flooding is less frequent, and at the upper limit of saltmarsh, it occurs perhaps only on very few occasions in a year during the very highest spring tides. The consequence of this pattern of flooding is rarely to establish a monotonic gradient in soil salinity from seaward to landward. At the lowest levels, the frequent flooding results in relatively stable salinities similar to those of the flooding water. As flooding becomes less frequent, the soil salinity is affected by rainfall that lowers salinity, and by dry periods when evaporation can result in hypersalinity [2]. Mangroves in general do not tolerate soil salinities much above that of seawater, but some, although not all, saltmarsh species can survive prolonged hypersaline conditions. Changes in temperature and rainfall are likely to alter the patterns (spatially and temporally) of soil salinity in the upper intertidal zone.

The coastlines on which saltmarsh and mangroves occur experience the full diversity of possible tidal regimes [24]. Tidal ranges vary from micro tidal to macro tidal (up to more than 10 m during spring tides). The tidal range will interact with sea level rise—the eustatic rise in ocean level produced by global warming will probably have greater biological consequences on micro tidal coasts, where the rise will be a significant proportion of the tidal range, than on macro tidal coasts where the same absolute rise will be a much smaller proportion of the tidal range [30]. Tidal range affects other environmental factors—the greater the range, the faster the tidal currents, which may dislodge newly germinated seedlings and restrict the window of opportunity for establishment to periods of neap tides; the greater the tidal range, the greater the depth to which low marsh communities are submerged and thus the greater the reduction of light during tidal flooding. In some localities, tidal fluctuations are less than the seasonal variation in the water level, for example, in the eastern Baltic and in the estuaries of southwest Western Australia [31]. Intermittently open coastal lagoons are tidal when the lagoon mouth is open (naturally or, as is increasingly the case, as a result of human intervention), but are non-tidal when the entrance is closed. The closed phase may last several years, but fringing saltmarsh persists during this period. Mangroves also occur in intermittently open lagoons, but less commonly than saltmarsh.

Beneath many saltmarshes and mangroves are freshwater aquifers, although the extent of interaction between the surface wetland and the underlying freshwater has been relatively little studied. Changes in the depth and flow rate within aquifers, as a result of greater extraction further inland, and the effects of climate change on both recharge and evapotranspiration, could have consequences for the intertidal vegetation.

## 3. Saltmarsh and Mangroves–A History of Use and Abuse

While climate change will have a major impact on saltmarsh and mangroves, the effects of climate change will interact with many other stressors acting on these ecosystems. Adam [23] suggests that while it is essential to address climate change issues, there are many other pressing management concerns which also require urgent attention. If these other matters are not dealt with then, even if the impacts of climate change are reduced, there may be few intertidal wetlands left to save.

Human exploitation and use of saltmarshes extends back centuries [19,32,33,34]. Some of the older uses such as livestock grazing and haymaking have declined in importance in recent years, but are still important to local economies in some areas. Reclamation of saltmarsh for agricultural use and to provide sites for development extends back to at least Roman times, but in the last century, there has been a rapid expansion of major developments of new or expanded ports, industrial and urban development, recreational uses and aquaculture [32,34]. There has been an equally long history of hunter-gatherer activity in mangroves, including collection of firewood and some logging (with one of the few demonstrated examples of sustainable intensive forestry in the tropics being practised in Malaysian mangroves for over a century [35]). Conversion of mangroves to aquaculture ponds has occurred on a large scale over the last few decades. Despite increasing international concern about the loss of mangroves to aquaculture the Brazilian government has recently permitted the shrimp fishing industry to convert extensive areas of intertidal wetlands into production ponds [1,36]. Absolute loss of intertidal wetlands has been accompanied by degradation of much of the remainder through pollution and numerous small-scale (but cumulatively large) disturbances from such causes as recreational bicycle use [2,32,33,34]. While the direct utilitarian values of coastal wetlands have been recognised by local communities for centuries, increased ecological understanding of the last 50 years has resulted in public opinion favouring measures to protect habitats for other reasons. The potential interactions between intertidal wetlands and adjacent waters have been seized upon by both professional and recreational fishers to justify habitat protection [37]. Interest in the conservation of intertidal wetlands began to develop at the start of the conservation age in the early 20th century, but was applied to sites that were recognised as special (some of the longest established formal reserves in a number of countries were saltmarshes). More general recognition of the importance of saltmarshes and mangroves leading to planning regimes designed to confer greater protection extends back to the 1960s [38], but despite great public support for such activities the continuing attrition of coastal wetlands has continued with many major developments being permitted because they were “in the national interest” which was deemed to override environmental concerns. The future of mangroves is particularly uncertain. Rapid urbanisation adjacent to many tropical estuaries, and the continuing expansion of aquaculture has resulted in considerable increase in the rate of mangrove loss [39,40]. The recent recognition of the amount of carbon sequestered in mangroves has led to the development of economic models for providing carbon credits for the retention of mangroves [41], (see also commentary [42,43]). Whether these mechanisms will be employed, and whether they can be successfully applied, is still to be determined.

Climate change may also, indirectly, create further impacts on coastal wetlands. For example, some of the most extensive saltmarshes in the world are in the Arctic [44]. Global warming may increase opportunities for human activity in locations that were inaccessible, permitting the damming of rivers flowing into the Arctic Sea for hydroelectricity generation, geological exploration, and the establishment of new hydrocarbon extraction projects and mineral mines. Shipping may also be able to more readily utilise Arctic waters during the summer. The environment will, however, still be challenging and the risk of accidents leading to widespread pollution will remain high. The ability of environmental regulators to control development on this new frontier will be challenged. The rate of environmental change consequent of global warming will be higher at high latitudes; if pollution and other disturbances add to the stresses of climate change the potential impacts on Arctic saltmarshes could be very great. Further, since the 1930s, annual freshwater discharge from the major Eurasian rivers flowing into the Arctic Ocean has been increasing [45]. The increase is correlated with changes in the North Atlantic Oscillation and with global mean surface air temperature [45], and it is suggested that the increase in freshwater flows may have important feedbacks to further changes in oceanic circulation and climate [45]. The freshening of Arctic coastal waters is also likely to have ecological consequences.

At lower latitudes potentially one of the indirect effects of climate change on saltmarshes and mangroves will be from attempted control of insect vectors of important diseases of humans and livestock. Diseases of concern include malaria and a large, and increasing, range of arboviruses [46]. The major vectors are mosquitoes, but nuisance insects, for which control is also demanded, include midges, sandflies and tabanids. In the United States, there has been a long history, dating to the early 20th century, of habitat modification to control insects in saltmarsh through drainage [2]. In Australia, a less destructive habitat modification, runnelling, has been applied to subtropical saltmarshes [47] and habitat modification of mangroves has recently been proposed by Knight [48]; traditionally, vector control in mangroves has relied on chemical treatment. Climate change may affect the distribution and abundance of disease agents, and of both primary and intermediate vectors [46]. Spread of mangroves into new areas may provide more habitat for some vectors. These factors, and the continuing increase of the human population close to tidal wetlands, will lead to greater control efforts. Use of chemicals may inflict collateral damage on other fauna. Use of habitat modification in mangroves remains to be tested, and while runnelling has been successful in floristically species-poor saltmarshes, whether it will prove to be as benign in more species-rich communities is as yet unknown.

## 4. Climate Change and Intertidal Wetlands

The possible effects of climate change will be complex and may vary even at sites relatively close together. These effects include those arising from:

- increases in ambient temperature
- changes in other climatic factors: rainfall, storms (including the frequency and intensity) and extreme events
- increases in sea level
- increases in atmospheric carbon dioxide concentration

### 4.1. Increasing Temperature

Many individual saltmarsh and mangrove species have very extensive distributions, with a number occurring in several continents. However, the latitudinal limits of species are often correlated with temperature (for example a number of European saltmarsh species share a common northern range limit [2] and along the eastern Australian coast the number of mangrove species declines with decreasing temperatures from tropical northern Queensland with more than 30 species to a single species in temperate Victoria).

It is a long established tenet of phytogeography [49] that the major factor determining the distribution of plant species is temperature. The influence of temperature is clearly seen in the distribution of many saltmarsh and mangrove species. Accordingly, we can expect the distribution of individual species to change with increased temperature, with species currently restricted to lower latitudes extending to higher latitudes. There have been relatively few studies addressing this expectation. For mangrove and saltmarsh species to move they need to possess traits which would permit movement of propagules, and, in particular, movement not just within single estuaries but between estuaries. Some (although not all) mangroves produce relatively large and buoyant viviparous seedlings. The large size means that the seedlings contain resources which could be sufficient for the seedlings to remain viable for long enough to be transported large distances. During the Quaternary, the range of mangrove species contracted, with high latitude populations becoming extinct because of cold and dry conditions. In low latitudes, populations may have become reduced because of the lack of suitable habitat when sea levels were lower [50]. Genetic studies have demonstrated recent long-distance dispersal of *Avicennia germinans* so the occurrence of this species on both sides of the Atlantic is due to dispersal and not to long-standing vicariance [50].

For saltmarsh plants there are two obvious mechanisms for movement. Many species have relatively small seeds and could be transported by birds. There is evidence that migratory waders and waterfowl do incidentally carry viable seeds [2,16]. Other seeds could be carried away from marshes by tides, and then dispersed by currents. Most mangrove propagules are relatively large and are unlikely to be dispersed by birds (in addition, they are not of a form which would be protected during bird transport, and wading birds do not generally frequent mangroves). For saltmarsh and mangrove propagules to be transported by currents, an important consideration is whether currents will carry propagules in the direction necessary to respond to warming temperatures. This may vary regionally, so that some populations might migrate and others will be trapped and decline, possibly to local extinction.

Mangroves are likely to respond to the combination of higher temperatures and higher carbon dioxide by spreading to high latitudes and growing more vigorously. Not only will there be a spread of mangroves species, but richness is likely to increase at high latitudes. On the east coast of Australia there may be an increase in species richness at high latitudes in response to climate change. The recent expansion of *Rhizophora stylosa* towards its southern limit of its range may be an early indication of response to climate change [2]. Expansion of mangroves in saltmarshes in eastern and southern Australia has been widely reported [2], and increased temperature may be one of the factors underlying this spread. Increasing temperature may also lead to higher incidence of fire in saltmarshes. Fire has long been used as a management tool in a number of regions, but wildfires also affect saltmarsh. Upper marsh rush and sedge communities are highly flammable, although recovery from fire may be slow. Mangroves are much less likely to burn, although local patch death can occur after lightning strikes. Changes in frequency of storms could result in more lightning strikes.

Future climate change will result in changes to components of fire regimes with the likelihood of increased frequencies and intensity of wildfires [51]. Intertidal wetlands might not immediately be thought of as fireprone are likely to be affected by changes in terrestrial fire regimes. However, tall saltmarsh vegetation, dominated by reeds, rushes or sedges, generate high fuel loads. In North America, there is a long history of use of prescribed fire for the management of saltmarshes for muskrat and nutria (coypu) [52,53], the improvement of waterfowl habitat [54], the increase of primary production through nutrient release, and the reduction of the impacts of wildfire [54]. Despite the historical use of fire, the effects on intertidal wetlands are poorly understood [54]. In northern Queensland (Australia) *Sporobolus virginicus* saltmarsh grasslands have been burned to promote the availability of new growth for grazing livestock [55]. It has been suggested [56] that if prescribed burning were discontinued, then accumulation of organic material, raising the marsh surface, would be promoted. The flammability of upper marsh vegetation means that, provided there is an ignition source, burning can occur. Fires can spread into saltmarsh from adjacent terrestrial communities, or result from accidental or deliberate ignition by humans. Wildfire in saltmarshes has been report from several localities in Australia [57,58] and has been observed (P. Adam, pers. obs) at a variety of locations in Australia and elsewhere.

Mangroves are much less likely to burn than saltmarshes (although in the major bushfires north of Sydney NSW in 1994 fires burning in terrestrial sclerophyll communities spread into mangroves in the Hawkesbury estuary; PA pers. obs), but local patch deaths following lightning strikes have been reported from mangroves in many localities [59,60,61,62,63]. The gaps are most commonly 10–20 trees in size and it is postulated that the likely cause of their being more than a single tree size is that when the central trees are struck, there is conduction of electricity to neighbouring trees through wet saline soil and because of the occurrence of root grafts [63]. Replenishment of the gaps through seedling establishment occurs. However, Duke [63] suggested that in the event of loss of trees from the seaward edge of stands because sea level rise exceeds the rate of replenishment, then more general collapse of stands could be expected. If there is greater storm activity and damage exceeds the rate of replenishment, then larger areas within stands could be lost. In addition to damage from lightning strikes, severe hailstorms have been reported to cause canopy damage [63,64]. An increase in frequency of severe hail might be one common consequence of the change climate.

### 4.2. Other Climatic Factors

Changes in rainfall distribution (temporally and spatially), could affect soil salinity, with consequent changes in vegetation composition.In south-eastern Tasmania increased temperature and wind speed and reduced rainfall since 1975 have been reflected in changes in saltmarsh vegetation composition and structure in favour of more salt tolerant communities [65]. Changes in the incidence of extreme events such as cyclones and hurricanes could result in damage to vegetation and erosion of intertidal wetlands. Intact mangrove and saltmarsh vegetation plays an important role in absorbing wave energy, thus protecting the hinterland from damage [66], but more frequent major storm damage could result in slow recovery and reduction in this protective role.

Storm surges, the temporary increase in sea level due to low atmospheric pressure and high wind can add up to several metres to the normal sea level [67] with wind, waves and swell compounding the impacts. This can result in inundation by seawater well beyond the normal tidal limit. Storm surges are normally of brief duration and, if the peak storm surge coincides with high tide, then the effects will be greater than if the peak is at the low tide (and the impact will be further influenced by the tidal range) [67]. Absorption of energy by saltmarsh and mangroves can reduce erosion and damage to the terrestrial zone adjacent to the intertidal wetlands [66,67], but a considerable width of wetland is required to have a significant effect [67]. Intertidal wetlands whose integrity has been compromised by human activity, including reduction in the binding of settlement by belowground biomass following eutrophication, may be more vulnerable to storm damage.

Wind-generated waves can cause damage to both saltmarsh and mangroves, but even extremely high winds are unlikely to damage the herbaceous vegetation saltmarsh. Strong winds can cause defoliation of mangrove canopies and break branches and tree trunks, damage which may remain visible for years after the impact. Mangroves may regenerate from propagules vigorously after major storm damage, but the seedlings and saplings may remain vulnerable to secondary damage when fallen tree trunks, moved by the tide, crush the young growth [62,63]. Rising relative sea level in conjunction with even small changes in frequency, intensity, timing and distribution of major storms is likely to have considerable impact on coastal wetlands and ecological processes [68]. It is likely during the 21st century that average tropical cyclone (cyclones, hurricanes, typhoons) maximum wind speed will increase, although it is likely the global frequency of cyclones will decrease or remain constant [69]. Predicting the occurrence and impacts of storms in particular locations is not possible, due to the complexity of both predicting the meteorological events and the site-specific factors which may influence response.

### 4.3. Change in Sea Level

Sea level has changed throughout geological history. The global sea level reached its approximate current position about 6,000 years ago, having risen from the last glacial minimum. Today’s saltmarshes and mangroves cannot have occupied their current sites for more than 6,000 years, and many are younger than this [2]. Nevertheless, saltmarsh and mangrove species are much older than 6,000 years, and many are restricted to these ecosystems, but have survived the changes in sea level, which would have required both vertical (10 s of metres) and lateral (in some regions, many kilometres) movement. Is there any cause for concern if the sea level changes in the future? In some parts of the world, such as Northern Australia, where most of the coastline is undeveloped, the response of coastal wetlands is likely to be similar to that which has happened in the past. However, humans have modified much of the coastline elsewhere and there are now many impediments which could limit or prevent wetlands moving in response to sea level rise. Global warming will create a eustatic rise in sea level by two mechanisms, thermal expansion (in which the oceans act as a giant thermometer) and through an increase in the amount of water as a result of melting glaciers and ice caps. This sea level increase will be global, but the effective change in sea level in any particular locality will be affected by a number of factors: eustatic, isostatic, tectonic and local [70]. Relative sea level will be the result of the interactions between these factors [70,71]. The isostatic component is due to the uplift of land as a result of deglaciation when the downward pressure exerted by the mass of ice is removed. The upward movement occurs over a much longer time scale than that of the retreat of the ice [71,72]. On tectonically active coasts, earthquakes can result in abrupt intermittent changes in relative sea level, with some lifting wetlands above the intertidal and others resulting in permanent submergence [2,33]. Local factors occur when changes in coastal geomorphology affect the tidal range. Change in tidal range will be experienced by many organisms as if they were changes in sea level. Various biological indicators are used to calculate past sea levels, so it is important that local events are recognized and discounted for purposes of these calculations [70]. In coastal lagoons, or inshore of chains of barrier islands, the tidal range is substantially dampened compared with that on nearby open coasts [70]. If inlets are widened or barriers breached in the course of coastal evolution or because of major storms or tsunamis, then local tidal range will increase. The interaction between these various factors results in considerable spatial variation in changes in relative sea level. The increase in volume of the oceans will not be expressed as a uniform increase in relative sea level along the world’s coasts.

Not only will there be geographic variation in future changes in relative sea level, but the response of intertidal wetlands will also be variable. Thus, while it will be possible to make generalised statements about wetland responses to rising sea level, application of these generalisations will need to reflect local circumstances, so that even sites in relatively close proximity will show differences in response. Semeniuk [73] developed conceptual models for the response of mangroves in northwestern Australia to sea level changes (while acknowledging that mangroves will also be affected by changes in the incidence of storms and increased temperature). He predicted that the response would vary with tidal range, geomorphological setting, local sedimentary regime, the influence of groundwater and the effects of changes in groundwater regimes (which were likely to be particularly important on the more arid sections of the coastline), species composition and biology, particularly whether recruitment was by seedlings or whether there would be responses largely through vegetative propagation. He suggested that heterogeneous coasts, characterised geomorphologically as rias, mangroves would not adjust to rising sea levels as quickly as homogeneous coastlines and there would be greater likelihood of disruption of mangrove communities. If sedimentation rates keep pace with or exceed rising sea level, intertidal wetlands could be maintained or even expand. Sedimentation may be through accumulation of carbon in the soil, or through the trapping and incorporation of inorganic material or by a combination of both. Mangroves have the potential to incorporate large amounts of organic material below ground and in addition, their complex aerial root systems may trap inorganic material. Saltmarshes may also incorporate organic material belowground; this is particularly the case with *Spartina* marshes, but in other types of saltmarshes, accretion may be driven by minerogenic sediment [4]. Given the special complexity of intertidal wetland response to rising sea level there is general agreement that within the range of predictions for sea level rise up to 2100 the majority of tidal wetlands will be able to adjust [30,74]. Alongi [74] suggests that a decline in the range of 10 to 15% in the majority of mangroves by 2100 in response to climate change is a realistic expectation. This loss would not be uniform and Alongi [74] identifies some coasts, particularly those with lower tidal ranges, that would be more vulnerable to loss. Both Alongi [74] for mangroves and Adam [75] for saltmarshes suggest that the more immediate threats from a range of other human impacts are likely to cause greater losses than climate change before 2100 [75]. Some of these factors may interact with the effects of climate change.

Eutrophication of estuaries and coastal waters is a global problem in temperate and tropical latitudes [76,77]. As a general rule, vascular plants respond to higher nutrient availability by allocating more of their productivity to shoots and leaves below ground [78]. This has been confirmed in studies in saltmarsh [78] and mangroves [79]. Less allocation below ground could limit the ability of sites to adjust to rising sea level, increase mortality because of stress during times of high soil salinity and low humidity [77], and increase the risk of erosion during storm events because fewer roots and rhizomes bind the sediment [80]. Eutrophication may not be the only explanation for the collapse of saltmarshes on the Atlantic coast of North America [80]. Localised depletion of top predators at sites accessible to recreational fishers has resulted in a great increase in populations of herbivorous crabs. This results in excess consumption of saltmarsh vegetation. It is postulated [81] that overfishing may be responsible for the widespread loss of saltmarsh due to high herbivore pressure. It will be difficult to separate the effects of climate change from other anthropogenic impacts on intertidal wetlands; from a coastal management perspective, all the impacts need to be managed.

Although eutrophication and the impact of overfishing will be widespread, there will also be continuing site-specific impacts, both from major industrial development (which, for the most part, will be assessed through planning systems) and numerous small *ad hoc* impacts. An investigation of changes over 25 years in saltmarshes in northwest England, a region where a number of species are close to their geographical limits of distribution (species reaching both northern and southern limits) was not able to detect any signals of climate change in the changed distribution of these species [75]. Despite the long-standing recognition of the conservation significance of the saltmarshes in this region, populations of species of interest have been lost through impacts such as upgrading of sea defences, trampling on both informal and formal footpaths, and construction of car parks.

If sediment accumulation, either from sand, silt and clay carried in by the tide or through autochthonous increase in soil organic matter, occurs at a rate equal to or greater than the sea level rise, then wetlands will be maintained or even expand. If sedimentation does not keep pace with the rise in sea level then wetlands will suffer loss extending upwards from their seaward edge. The greater the eustatic sea level rise, the greater the probability of wetlands loss, but in the short to medium term, many coastal wetlands may be able to keep pace with sea level rise (see various chapters in reference [82]). If intertidal wetlands do not keep pace with rising the sea level then erosion will occur from the seaward margin and the community will potentially retreat landwards. In some locations, natural topography may limit the extent to which retreat will be possible, but more frequently, artificial barriers will prevent movement landward. If retreat, for whatever reason, is not possible, the area of intertidal wetland will decline, the victim of “coastal squeeze”.

Where saltmarsh and mangrove coexist, expansion of mangroves upwards may also squeeze out saltmarsh. For a number of decades, mangroves have been expanding into saltmarsh at sites along several thousands of kilometres of coastline from the Sunshine Coast in Queensland to western South Australia [83], in a way which is compatible with a response to a sea level rise scenario. Sea level rise may be a factor in the expansion of mangroves experienced over the last few decades, but other factors including variation in rainfall, temperature and increased carbon dioxide could also have affected mangrove growth and competitiveness. In southeast Queensland, it has been shown that the rate of mangrove expansion into saltmarsh was higher during years of high rainfall [84], for which possible explanations include reduced salinity and increased nutrient and sediment loads from greater catchment runoff. The dynamics of mangrove and saltmarsh interactions outside of southeast Australia have been less well studied. A similar phenomenon occurs in the North Island of New Zealand, but in the southern United States of America, there have been periods of mangrove expansion (by *Avicennia germinans*) into saltmarsh followed by rapid recession after mangrove death as a result of frosts. Recently, following drought-induced saltmarsh death, there has been a period of more extensive mangrove establishment into former saltmarsh [83]. Declining incidence of frost could lead to greater expansion of mangroves.

Regardless of the relative contribution of different factors in the recent past, what has been observed is a foretaste of what will happen to a greater extent in the future. As both saltmarsh and mangroves are regarded as having high conservation value, the change in relative extent of the two ecosystems raises dilemmas for managers—should there be changes permitted to take their course or should there be active intervention?

### 4.4. Increase in Carbon Dioxide Concentration

The increase in atmospheric carbon dioxide concentration will have a number of impacts on coastal wetland ecosystems. The fixing of carbon dioxide in photosynthesis is one of the key global ecosystem processes. Increasing carbon dioxide will lead to higher plant productivity, but not all plants respond equally. Mangroves, being trees, are C_3_ species and physiological [85], anatomical [14] and isotopic evidence [86] confirms this. Two major families in saltmarshes worldwide are the Poaceae and Chenopodiaceae. Several dominant grasses in saltmarsh, notably *Spartina* species and *Sporobolus virginicus* are C_4_ species: although the C_4_ pathway has evolved in a number of lineages within the Chenopodiaceae [87], the major genera and species which occur in intertidal salt marsh are not C_4_. Many saltmarsh chenopods are succulent, but succulence is associated with tissue salt regulation and not possession of the CAM (Crassulacean acid metabolism) photosynthetic pathway [2]. Halophytic chenopods are, however, of frequent occurrence in inland saline semi-arid and arid habitats.

Plants with C_4_ photosynthesis are relatively favoured under low atmospheric CO_2_ and/or higher temperatures [88]. In the future, both temperature and CO_2_ are expected to increase, imposing conflicting pressures on the competitive interaction between C_3_ and C_4_ species [88]. However, as CO_2_ levels rise above a threshold, C_4_ plants may lose their competitive advantage under the anticipated increased temperature regime [88].

Increasing temperature and carbon dioxide may be factors underlying the spread of mangroves into the saltmarsh currently occurring in some regions, but studies of the effects of increasing CO_2_ on mangroves show variation between species [74,89]. Mangroves in general have high water use efficiency [90], which may be an adaptation to minimise water uptake from saline soils, and water use efficiency may be increased by the elevated CO_2_ [91]. Water use efficiency increases due to reduced stomatal conductance at high salinity which will reduce carbon dioxide uptake and result in lower assimilation rates [89]. Thus the responses of individual mangrove species to climate change are likely to reflect complex interactions between a number of factors [89].

Saltmarshes in temperate and tropical regions are mixtures of C_3_ and C_4_ species. The dominance of C_4_ *Spartina* species in northern hemisphere marshes represents the highest latitudes at which C_4_ dominance occurs in any ecosystem [2]. Under increased temperature and higher carbon dioxide the balance between C_3_ and C_4_ species might be changed. In a long-running mesocosm experiment in the United States exposing saltmarsh to artificially enhanced carbon dioxide levels, C_3_ sedges increased at the expense of C_4_ *Spartina* [92,93,94], possibly indicating what might be expected over larger areas some decades hence. At higher carbon dioxide levels, plants photosynthesise more efficiently, with higher water use efficiency. This results in lower rates of transpiration, so the increase in soil salinity between episodes of tidal flooding during summertime may be reduced. At higher carbon dioxide concentrations, the concentration of protein in foliage will be lower, thereby affecting the quality of food for herbivores. The effects of this will be experienced throughout the whole community; whether the effect will be to reduce the number of herbivores or to maintain herbivory by increasing consumption per individual is still to be determined, as has been suggested in a number of studies on *Eucalyptus* forests [95,96,97]. Changes to the nutritional status of foliage might have ecosystem scale consequence. However, predicting the effects of increased carbon dioxide on halophytes is complicated by the use by many species of nitrogenous compounds for osmoregulation within cytoplasm [2,98,99].

## 5. Climate Change and Fauna of Intertidal Wetlands

Saltmarsh and mangrove forest fauna may respond to climate change by shifting in distribution and abundance landward, evolving, or becoming extinct [100]. The capacity of fauna to move and adapt will depend on their life history characteristics, phenotypic plasticity, and the genetic variability and inheritability of relevant characteristics [101]. As Holt [101] remarks, predicting which will occur is difficult because there is almost no species where we know enough about its ecology, physiology, behaviour or genetics to predict its response. For example, it is difficult to derive reliable interpretations of long-term changes in saltmarsh fauna in southern hemisphere saltmarshes from the past literature because of the uncertainties generated by recent major taxonomic and nomenclatural revisions affecting many taxa [102]. Species that were once grouped together have become split and reclassified. The lack of attention to saltmarsh and mangrove forest fauna and the potential impacts of climate change is alarming given the role these ecosystems have in relaying nutrients and energy to coastal waterways [103] and as a nursery habitat for economically important fish.

Like many ecosystems, there is a scarcity of long-term ecological databases for intertidal wetland fauna [11,12,100] that can be used to detect observed responses to recent climate change. There are, however, long-term datasets on saltmarsh waterfowl and waders which can be used to document the impacts of habitat destruction and to detect long-term changes that might result from climate change [104]. There is also limited data on the physiological tolerance of fauna in intertidal wetlands to even basic environmental parameters such as temperature and salinity [105]. Unsurprisingly, predicted responses of saltmarsh and mangrove fauna to climate change are missing from many major reports, especially in the southern hemisphere [106,107], with the majority of commentary on predicted shifts in distribution of fauna relating to the northern hemisphere [108].

## 6. Acidification and Temperature Rise; Implications for Fauna

Ocean pH is now 0.1 units less than it was in pre-industrial times [109] and a further 0.3–0.4 unit decline is expected by 2100, and a 0.7–0.77 decline by 2300 [109,110,111]. This is a rate of acidification greater than what has previously been experienced by marine organisms [111,112,113], thus leading to uncertainty about whether fauna will be able to adapt [114]. At the same time as oceans acidify, they will warm. It is predicted that sea-surface temperatures will increase between 2–4.5 °C [115]. Although it is uncertain whether pH in coastal systems mirror changes in open systems, the likelihood of severe consequences for marine and estuarine biodiversity from ocean acidification is high [114]. Further, given the major role of temperature in controlling distribution and abundance, physiology, morphology and behaviour of marine organisms [116], the combined impact of warming and acidifying oceans, together with other synergistic and multiple stressors will be major factors in the sustainability of intertidal wetland fauna [117].

## 7. Life History of Saltmarsh and Mangrove Fauna

Life history is a key factor in determining whether fauna will be able to move to more suitable habitats. Many of the marine macroinvertebrates found in saltmarshes and mangrove forests are broadcast spawners, with fertilisation occurring in the water column followed by planktotrophic larval development. Larvae spend days to weeks in coastal waters in direct contact with potentially acidified and warmed oceanic waters. Following a period of time, late-stage larvae return to mangrove forests and saltmarshes as an important determinant of settlement, recruitment and adult distribution and abundance. Like propagule and larval supply on coastal rocky habitats, larval supply and habitat selection is an important determinant of adult distribution and abundance in saltmarsh and mangrove forests [118,119,120]. Fauna with pelagic adult or larval stages have been shown to respond to climate change by moving poleward [10,121]. Larval stages are predicted to be particularly susceptible to the combined synergistic stressors of temperature and acidification in saltmarsh and mangrove forests [117,122]. In contrast, sessile organisms without dispersive larvae which rely on asexual reproduction or direct development may be unable to move. Whether species shift in distribution and abundance, go extinct, or evolve will largely depend on their life history, their physiological tolerances, and behavioural capacities, and whether they have a restricted habitat preference. Species with a restricted range, such as many of those in saltmarshes, are more vulnerable to extinction than species which extend into mangrove forests and/or seagrass. In these later instances, if saltmarsh is missing then species may still survive with a reduced distribution and perhaps abundance. How alterations in climate will impact on the complex dynamics of larval recruitment is unknown, in part because of the absence of long-term datasets. Menge *et al.* [121] found, using a long-term data series, that phytoplankton abundance and mussel recruitment were positively correlated. Although larval food was suggested as a driver of mussel recruitment dependent on oceanographic and atmospheric fluctuations, this was potentially modified by local ecological interactions. Menge *et al.* [121] concluded that it is difficult to determine how climate change will alter the threshold level at which an ecosystem may shift to an alternative form, but that investigating the actual mechanisms that underlie ecosystems was still important. For saltmarsh and mangrove fauna, many of which have pelagic planktotrophic larvae, there is a need to better understand their range of responses to ocean acidification and temperature and the synergistic effect of other anthropogenic stressors [117,122].

## 8. Molluscs

A major group of calcifying macroinvertebrates found commonly in saltmarsh and mangrove forests across the globe are the molluscs [81,105,123,124,125,126,127]. Both bivalve and univalve molluscs (bivalves and gastropods) are permanent residents of saltmarsh and mangrove forests as adults, with external shells in close contact with the sediment or structural features of the habitat. Being broadcast spawners, the early life history stages of fertilisation and larval development are susceptible to increased temperature and elevated CO_2_. Larval shells are comprised of either amorphous calcium carbonate (ACC) or soluble aragonite [128], making it potentially more difficult to calcify in an acidified environment. Reductions in calcification of adults in response to acidification and/or temperature have been found in a wide range of species [129,130,131,132,133], but not all [134,135] (Table 1) and their larvae [136,137,138,139,140,141,142,143,144,145,146,147,148,149,150,151]. Larval stages of commercial species of estuarine oysters, including *Crassostrea gigas*, *Crassostrea virginica* and *Saccostrea glomerata* [131,141,142,143,144,146,147,152], and mussels [145], have been found to be particularly vulnerable to ocean acidification, with a reduction in size, increase in abnormal development, a delay in development time, and a reduction in larval survival [132,141,142,143,144,146], although whether early stage larvae are more sensitive than later-stage larvae is still questionable [153]. Talmage and Gobler [154] also found that the lipid content of larvae decreased and shell dissolution increased with increasing CO_2_. Any compromise to energy reserves and shell integrity of larvae may make them more susceptible to predation and affect metamorphosis, settlement and survival [155,156,157,158,159]. Indirectly, responses of gastropods to produce thicker shells in the presence of cues from predatory crabs can be altered by acidification [156], cause a reduction in metabolism and energetics affecting other processes such as reproduction and growth [116], making molluscs more susceptible to disease [160]. Given that mangrove and saltmarsh gastropods are abundant and significant as detritivores across the globe [81,105,123,124,125,126,127], relaying nutrients and energy from the intertidal to adjacent coastal waterways [4,5,6,103], any alteration in fertilisation, larval survival and reduction in fecundity and growth of adults may have flow on effects to the surrounding ecosystems such as seagrass and coastal fisheries. Saltmarshes and mangrove forests can be dominated by mussels, oysters and gastropods in hundreds to thousands of individuals per square metre [81,105,123,124,125,126,127,161]. In southeastern North American saltmarshes, plant biomass and production of *Spartina alterniflora* are controlled by the grazing of the periwinkle, *Littoraria irrorata*, and its predators such as blue crabs, *Callinectes sapidus*, in a trophic cascade [124,125,126,127]. Top-down control of grazer density regulates saltmarsh plant growth and overharvesting of blue crabs that are snail predators may lead to increased die-off of saltmarshes [81,124,125,126]. Ocean acidification has been found to dissolve shells of gastropods and mussels, making them more susceptible to predation [157] and may disrupt trophic cascades [124,125,126] because gastropods with weakened shells will be more susceptible to the crushing by crabs. The fauna of intertidal wetlands, including molluscs, also create habitat for other organisms and provide refuges from predation [123]. Factors which weaken or alter these structural elements (Table 1) and change species compositions, *i.e.*, between C_3_ and C_4_ vegetation [92,93,94], may also affect recruitment, mortality and growth of other organisms in complex ways. Warming will also have significant impacts on molluscs. Warmer seasons may have positive or negative impacts, depending on the species of interest. Some species may expand their distribution to higher latitude, while species originally occurring at higher latitudes may retreat to even higher latitudes [10,101,162]. Increased temperature may stimulate phytoplankton productivity, leading to greater consumption by zooplankton, with a net effect of less phytoplankton being available for filter feeding molluscs [162]. Acidification and warming may also act in synergy with other stressors such as eutrophication, recently found to have a role in the dieback of saltmarshes [80] and decrease oxygen solubility (increase hypoxia), which may have implications for the ecological interactions of molluscs in both hemispheres. Sea level rise may also allow movement of more predators upstream [162]. The only study so far to investigate resilience of estuarine oysters to climate change [163] found there may be a capacity for adaptation to climate-induced acidification, whereas other forms of acidification in estuaries reduced oyster growth, but did not affect mortality [158,159]. Perhaps the best-known oyster larval mortalities from acidified seawater have been along the Pacific Northwest of America, where the lack of sufficient spat has threatened an industry worth US $278 million in 2009 [146,152]. Further evidence that other calcifying fauna in intertidal wetlands may be affected by climate change, is provided by the research on calcifying larval echinoderms, which have found in laboratory studies to be very susceptible to elevated CO_2_ and temperature [164,165,166,167,168,169,170,171,172,173,174,175,176,177,178].

## 9. Crabs and Crustaceans

On all inhabited continents, saltmarshes and mangrove forests are important habitats for crustaceans, especially crabs as they are economically valuable [179,180,181,182,183,184,185,186]. Generally, crab diversity and densities increase with decreasing latitude [180,181] with temperature rather than acidification being more likely to influence species distributions. In both the northern and southern hemispheres, crabs have a role in modifying mangrove and saltmarsh habitats [180,181,182,184,185,186,182,184]. As a result of their borrowing, they can oxygenate the sediment, alter nitrogen and chlorophyll levels, and enhance productivity [181,182,183,184,185]. Like molluscs, in saltmarsh habitats, crabs contribute substantially to nutrient cycling and energy flow [187] and crab larvae are an important food source for fish.

In the studies to date on barnacles [188,189,190,191], crabs [192], amphipods [193] and other crustaceans [194,195] (see Table 1), there have been few significant impacts of ocean acidification detected, although the effect of acidification was stronger than temperature at the northern edge of the range of *Semibalanus balanoides* barnacles, perhaps slowing down a northern expansion of its range [191]. Walther *et al.* [192] investigated the combined effects of elevated CO_2_ and temperature on two populations of the cold eurythermal spider crab *Hyas araneus*. At elevated CO_2_ conditions, there was a lower capacity for calcium incorporation in crab larvae from the colder northern most (79° N) compared to southern populations (54° N). This study provides evidence that species responses to ocean acidification and warming may vary across their range with greater sensitivity in colder regions.

Depletion crabs which are top predators in saltmarshes may also alter ecological processes and lead to a proliferation of other herbivorous crab species and gastropods which may result in widespread loss of saltmarsh due to high herbivore pressure [81,125,126]. It is likely that there will be some degree of range shift in the distribution of adults if the findings of Walther *et al.* [192] and Findlay *et al.* [191] are transferrable to wetland fauna, and if temperature, rather than acidification, is more important in these crustaceans with calcium phosphate exoskeletons. Most studies to date on the impacts of ocean change have focused on calcifying marine organisms due to concerns of reduced aragonite and calcite saturation [128,196] (Table 1). It is more likely, however, that ocean acidification and temperature will act together [122,136] with ocean acidification becoming more significant if the thermal tolerance of the organism is breached [116]. More data is needed, especially on the synergistic responses and the thermal tolerance across a range of ontogenic stages for crabs and their larvae, given the significant role of larval crabs as a food source for fish in estuaries and wetlands [197,198,199,200,201,202,203,204,205].

**Table 1:** The impacts of ocean acidification and temperature on estuarine organisms and molluscs and crustaceans.

Species, habitat and distribution	Life history stage tested	Elevated *p*CO_2_ conditions (pH)	Impacts of reduced pH	Reference
**Molluscs**				
*Saccostrea glomerata* Existing substrata in shallow and intertidal estuaries of Eastern Australia	Larvae	6.75	Reduced development; increased abnormality	[159]
	Egg, larvae	7.9	Reduced fertilization; reduced larval growth and development; increased abnormality and mortality.	[141]
	Larvae	7.6	Reduced growth and development; increased abnormality and mortality.	[142]
	Egg, larvae	7.9	Reduced fertilization; reduced larval development and growth; increased abnormality	[143]
	Spat	7.84	Reduced growth; greater resilience of selectively bred line *vs.* wild population.	[144]
*Crassostrea gigas* Existing substrata in shallow and intertidal estuaries of Japan; introduced to Australia, New Zealand, and America	Egg, larvae	7.4	Fertilization unaffected; reduced growth and larval size; increased abnormality	[138]
	Egg, sperm	7.8	Fertilization unaffected; sperm speed and motility unaffected	[140]
*Crassostrea virginica* Formed reefs in subtidal and intertidal areas of North and Central America.	Larvae	6.25	Reduced growth; increased mortality	[147]
	Larvae	8.16	Reduced growth and calcification	[130]
	Larvae	7.50–8.07	Reduced growth; delayed metamorphosis; increased mortality	[154]
	Juvenile	7.5	Reduced growth; increased abnormality and mortality; increased standard metabolic rate	[131]
*Mytilus edilus* Existing strata in intertidal areas of the northern Pacific and Atlantic	Larvae	7.6–7.8	Reduced shell size; pH 7.6 reduced hatch rate	[145]
*Mytilus galloprovincialis* Intertidal regions of existing strata with high water flow; endemic to the Mediterranean.	Egg, larvae	7.4	Delayed metamorphosis; increased abnormality; decreased growth	[139]
*Argopecten irradians* Sandy subtidal beds in protected areas; endemic to Northern America	Larvae	7.5–8.17	Decreased growth, rate of development and metamorphosis; increased abnormality and mortality	[154]
*Cavolinia inflexa* Pelagic warm waters	Larvae	7.51–7.82	Increased abnormality; reduced growth	[133]
*Haliotis coccoradiata* Subtidal rocky strata of Eastern Australia	Egg, sperm	7.6–7.9	No significant effect on fertilization	[165]
	Larvae, juvenile	7.6–7.8	Reduced calcification and development; increased abnormality	[117]
*Haliotis rufescens* Subtidal rocky strata of North America	Larvae	7.87–7.97	Reduced thermal tolerance at pH 7.87 in pre-torsion and late veligers; no effect on post-torsion and premetamorphic veligers; no effect on shell mineralization genes *ap24* or *engrailed*	[148]
*Laternula elliptica* Soft substrates of the Southern Ocean	Adult	7.78	Basal metabolism and heat shock protein *HSP70* expression increased. Expression of chitin synthase significantly up-regulated	[149]
*Limacina helicina* Pelagic Arctic waters	Juveniles	7.78–8.21	Increased abnormal development, shell degradation & mortality	[151]
*Littorina obtusata* Intertidal rocky shores	Egg, larvae	7.6	Reduced viability and increased abnormal development	[150]
*Mercenaria mercenaria* Subtidal soft substrates in North America	Larvae	6.25	Increased mortality	[147]
	Larvae	7.5–8.17	Reduced rate of growth and development with increasing *p* CO_2_; increased abnormal development and mortality	[154]
*Sepia officinalis* Pelagic waters of Europe	Juvenile	7.1–7.23	Growth and development unaffected	[134]
	Egg	7.6–7.85	Egg weight increased; hatchling size unaffected; accumulation of silver increased, cadmium decreased; zinc accumulation highest at pH 7.85.	[135]
**Arthropods**				
*Amphibalanus amphitrite* Naturally found in intertidal regions, and has spread across the globe by attachment to ships	Larvae, cyprid	7.4	No significant effects	[189]
*Semibalanus balanoides* Intertidal regions of European coasts	Egg	7.7	Reduced rate of development in embryos	[188]
	Post-larval	7.7	Reduced calcification and survival in synergism with temperature increases	[190]
*Elminius modestus* Intertidal regions of European coasts	Post-larval	7.7	Decreased rate of growth	[190]
*Acartia tsuensis* Pelagic regions of the temperate Pacific	Egg, larvae	7.31	No significant effects	[194]
*Calanus finmarchicus* Pelagic North Atlantic	Egg	6.95	Reduced rate of hatching	[195]
*Echinogammarus marinus* Pelagic, most of the world’s oceans	Egg	7.5	Reduced rate of embryonic development	[193]
*Hyas araneus* Below low tide of the North Atlantic	Larvae	unknown	Delayed development; reduced rate of growth and fitness	[192]

## 10. Fish

Saltmarshes and mangroves are important habitats for fish, many of which are economically valuable [197,198,199,200,201,202,203,204,205]. General patterns are best described in northern hemisphere saltmarshes where 90% of research on fish was undertaken. In southern hemisphere saltmarshes, fish can reside only transiently at the highest of spring tides and studies in the southern hemisphere represent only 3% of the total number of studies reported on fish utilisation of saltmarshes [198]. Despite the relative low frequency of tidal inundation of saltmarshes in some areas of the globe, both saltmarshes and mangrove forests act as nursery grounds and resources for fish [197,198,199,200,201,202,203,204,205,206,207,208]. Relationships between the size of wetlands and connectivity among the estuarine mosaic and weight of harvested fish from both hemispheres emphasise the importance of estuaries for the nutrition of fish [198,205,206,207,208].

Despite the fact that economically significant fish use wetlands as nurseries and provide ecosystem services which may extend beyond their boundaries, we have a substantial knowledge gap on the effects of ocean acidification or global warming on fish. Fish may be better than osmo-conformers than macroinvertebrates in regulating their acid-base status to compensate for acidosis, but fish have been found vulnerable to ocean acidification, demonstrating impaired olfactory discrimination and homing ability [209] and a reduced capacity to avoid predators [210]. Although studies have found that mortality of larval and adult fish and fertilisation and egg survival appear tolerant of high CO_2_, these results are based on a limited number of coral reef species. Changes in the capacity of fish to detect saltmarsh and mangrove forests may alter the nutritional role that these habitats have for juvenile fish at high tide [209,210,211,212,213,214] and have flow on impacts for birds [215].

## 11. Avifauna

Saltmarshes and mangrove forests are important to birds providing sites for feeding, roosting and nesting [104,215,216,217,218]. A variety of birds directly eat the seeds and filamentous green algae. Increasing temperatures associated with climate change could alter a range of variables such as primary productivity and nutrient cycling which may affect abundances of macroinvertebrates with flow on impacts on birds both in terms of feeding and reproducing [215,216,217,218]. For example, the loss and degradation of Arctic saltmarshes from climate change is predicted to have global consequences for avifauna. Many species of wading birds currently nest in the Arctic and utilise food resources in saltmarshes and mudflats [104]. During the non-breeding, season these waders may be found in estuaries far to the south, not just in the temperate northern hemisphere, but also south of the equator. Bar-tailed godwits, for example, make marathon flights from Australasia to Alaska [217]. Migratory waders are vulnerable to the effects of climate change throughout their flyways, but declines in breeding success in the Arctic would be particularly serious for long-term survival of species. Rise in relative sea level may also reduce the area of intertidal mudflat, the essential feeding habitat for waders, and these losses in addition to the losses due to port and industrial development may result in substantial declines in populations of some species [218].

In the northern hemisphere, migratory waterfowl (duck, swans but particularly geese) also commute between Arctic saltmarshes and temperate regions (there is no equivalent phenomenon in the southern hemisphere). Changes in agricultural practices leading to greater availability of food from agricultural crops in the wintering grounds have permitted a huge increase in the population of several species of geese. Trampling and grubbing up plants causes substantial damage to saltmarshes in the Arctic breeding grounds [44] and there is also increased grazing pressure on temperate saltmarshes in the overwintering grounds as the birds do not spend all the time on agricultural land. Continued increases in waterfowl populations will further increase pressure on the breeding grounds, potentially exacerbating the effects of climate change.

## 12. Conclusion

Climate change is predicted to have a major impact on intertidal wetlands. Changes in climate may increase the rate of evaporation from the soil surface; increase or reduce salinity, depending on evaporation or precipitation; increase sea level; acidify waters; and, increase temperature. While some of the hypotheses proposed in the 1960s explaining the role of the estuarine ecosystem are now viewed as simplistic, the new synthesis [219] and availability of new techniques of isotopic analysis to trace material and energy flows [3,4,5,6,185,186] demonstrate that saltmarshes and mangroves do indeed play an important role in sustaining estuarine and coastal fisheries, and there are also important geochemical exchanges involving intertidal wetlands [220,221]. We know the least about organisms in southern hemisphere saltmarshes and mangroves. We know relatively little about fungi, for example, and terrestrially derived fauna (insects, spiders, *etc*.) have been studied at very few locations. Predicting what will occur is difficult because there is almost no species where we know enough about their ecology, physiology and behaviour. Nevertheless, there is more than adequate information to justify claims that, from a conservation perspective, saltmarshes are globally significant ecosystems which are increasingly being considered economically valuable [222], and blue carbon sinks [223] where climate change is likely to have a significant impact.
